# Infection Related Inferior Alveolar Nerve Paresthesia in the Lower Premolar Teeth

**DOI:** 10.1155/2016/2623507

**Published:** 2016-08-11

**Authors:** Rachele Censi, Virna Vavassori, Andrea Enrico Borgonovo, Dino Re

**Affiliations:** ^1^Department of Implantology and Periodontology, Istituto Stomatologico Italiano, 20121 Milan, Italy; ^2^School of Oral Surgery, Department of Oral Rehabilitation, Istituto Stomatologico Italiano, University of Milan, Milan, Italy; ^3^School of Oral Surgery, Policlinico, Fondazione IRCCS Cà Granda, University of Milan, Milan, Italy; ^4^LUdeS Foundation HEI, Valletta, Malta; ^5^Department of Oral Rehabilitation, Istituto Stomatologico Italiano, 20121 Milan, Italy

## Abstract

*Introduction*. The aim of this paper was to describe two cases of IAN infection-induced paresthesia and to discuss the most appropriate treatment solutions.* Methods*. For two patients, periapical lesions that induced IAN paresthesia were revealed. In the first case, the tooth was previously endodontically treated, whereas in the second case the lesion was due to pulp necrosis.* Results*. For the first patient, a progressive healing was observed only after the tooth extraction. In the second patient, the paresthesia had resolved after endodontic treatment.* Conclusions*. The endodontic-related paresthesia is a rare complication that can be the result of a combination of etiopathogenic mechanisms such as mechanical pressure on the nerve fibers due to the expanding infectious process and the production of microbial toxins. Paresthesia resulting from periapical lesions usually subsides through elimination of infection by root canal treatment. However, if there are no signs of enhancement, the immediate extraction of the tooth is the treatment of choice in order to prevent irreversible paresthesia because it was demonstrated that there is a correlation between the duration of mechanical or chemical irritation and the risk of permanent paresthesia.

## 1. Introduction

Paresthesia is a neurosensitivity disorder characterized by a burning or twinging sensation or by partial loss of local sensitivity and, in the literature, the causes of inferior alveolar nerve paresthesia are divided into systemic diseases and local factors [1]. Systemic causes refer to multiple sclerosis, sarcoidosis, viral and bacterial infections, metastasis, drug-induced diseases, and blood diseases [2]. Local factors correspond with mechanical, thermal, or toxic injuries of IAN. Mechanical injuries of the nerve include compression, stretching, partial or total resection, and laceration that are frequently caused by block anesthesia, third molar surgery, or local tumor infiltration [3]. Chemical trauma can be due to toxic components of different materials, such as the endodontic filling materials (paraformaldehyde), irrigating solutions (sodium hypochlorite), or local anesthetics. Thermal injury is a consequence of bone overheating during the performance of surgical techniques. Endodontic-related paresthesia is a very rare complication in dentistry and can be related to periodontal pathology (periapical lesions) or endodontic iatrogenic causes as a consequence of the filling material in the mandibular canal or overinstrumentation [4, 5]. In the majority of the cases reported in literature, the paresthesia involves the inferior alveolar nerve (IAN) and its branches and the duration of the neurologic symptoms varies greatly from days or weeks to several months and, in some cases, paresthesia might even become permanent [6]. The aim of this paper is to describe two cases of IAN infection-induced paresthesia and to discuss the most appropriate treatment solutions.

## 2. Case Report

*Case  1*. On November 2, 2011, a 41-year-old female patient presented to our office with severe pain in the lower left second premolar (#35) and swelling lower lip with extended paresthesia in the surrounding area. The tooth was treated about 10 years ago, restored with a metal ceramic crown, and never gave problems before.

Clinical evaluation revealed painful tumefaction on palpation in the buccal part of sulcus along the tooth, as well as lingual part in projection of the root, together with slightly red, inflamed gums. Paresthesia extension was explored with a probe and took part around the infected tooth, left corner of lower lip, and the chin.

**Figure 1:**
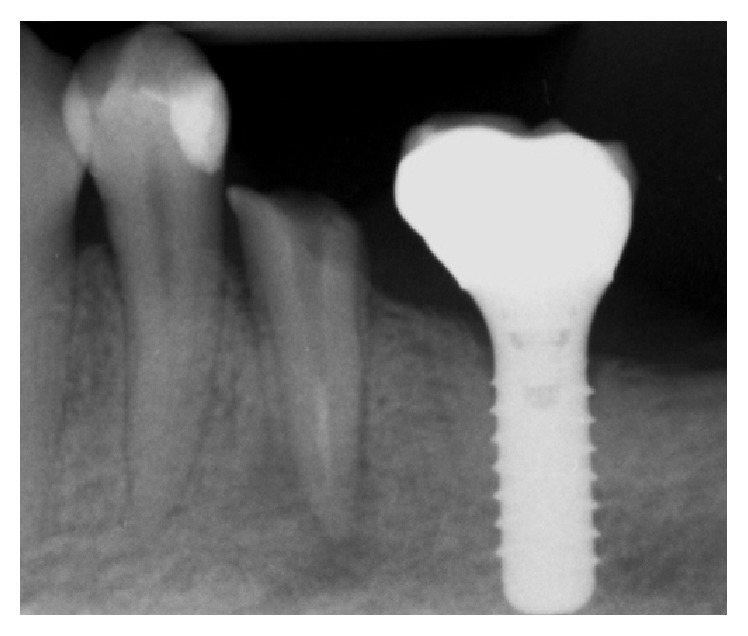
Lower left second premolar with apical lesion in contact with mandibular nerve canal.

Radiographic (OPTC) exam showed poor previous endodontic treatment. Moreover, a wide apical lesion in contact with mandibular nerve canal was discovered (Figure 1).

Antibiotics therapy was prescribed (amoxicillin/clavulanic acid, 1 g every 8 h for 7 days, and Metronidazole, Flagyl, Pfizer, 500 mg every 8 hours for 7 days). When the patient returned after 10 days, symptoms for abscess disappeared, but the numbness was still present.

**Figure 2:**
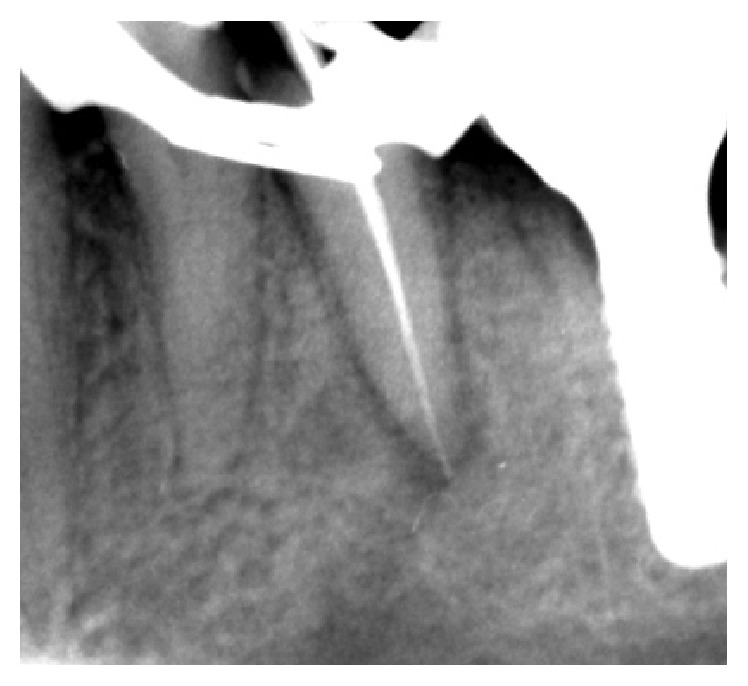
Endodontic retreatment.

**Figure 3:**
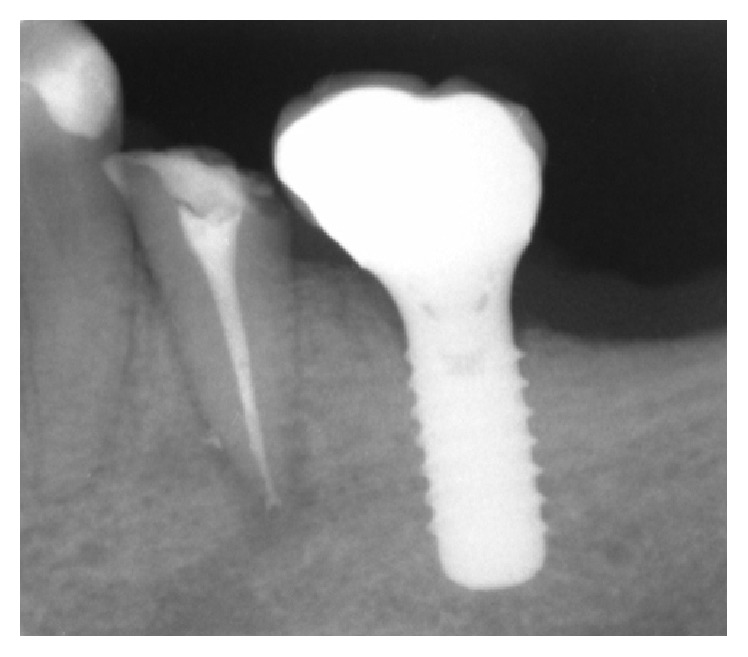
Radiographic control after endodontic treatment.

**Figure 4:**
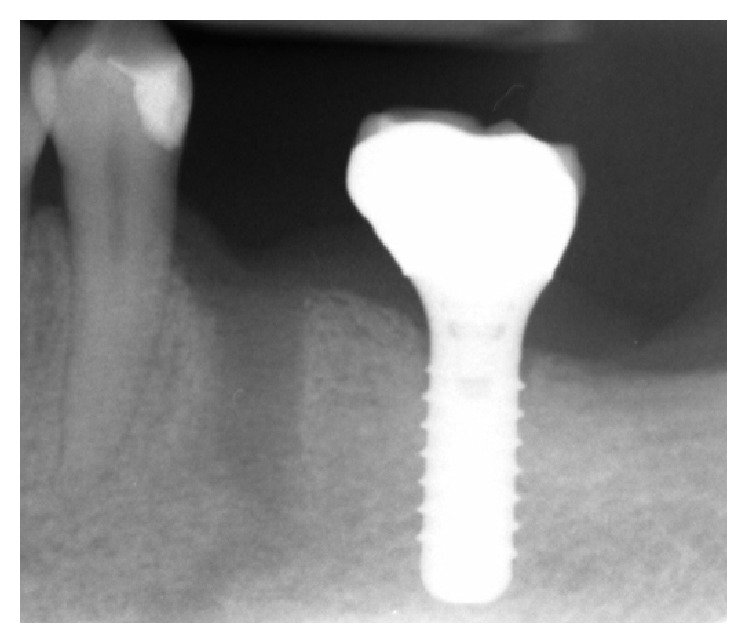
Radiographic exam after tooth extraction.

**Figure 5:**
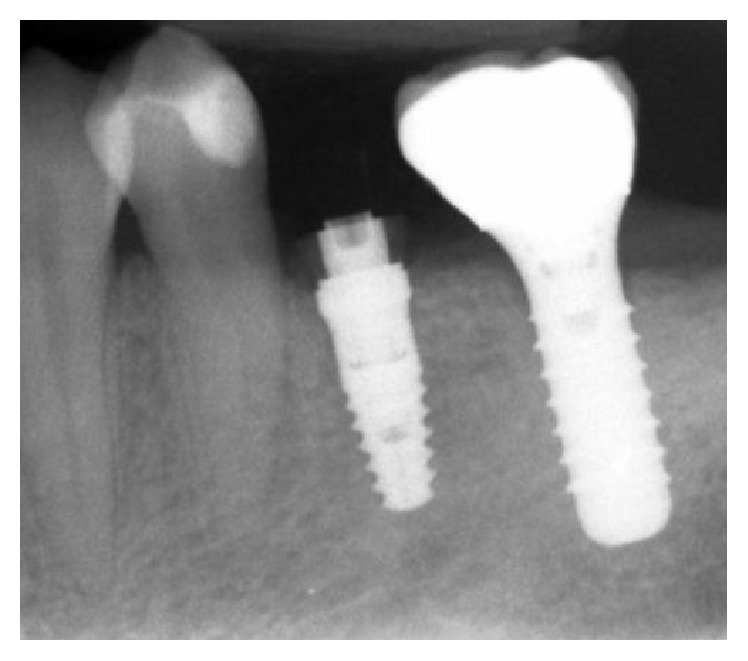
Implant rehabilitation.

Since the patient's desire was to try to maintain the tooth, endodontic retreatment was performed. With great caution to avoid overinstrumentation, present gutta-percha in the root canal was removed using GPR (gutta-percha remover, OGNA, Laboratori Farmaceutici) and shaped with rotatory and manual instrumentation. The canal was irrigated with sodium hypochlorite (NaOCl) finishing with #25 as a last apical instrument (Figure 2). Intermediate medication was not done using calcium hydroxide. This intracanal medication is a strong alkali and is able to cause permanent nerve damage although these cases are rarely described in literature [7]. When the patient returned for her scheduled appointment, the canal was filled with gutta-percha. Radiographic control examination showed no extension of filling material over the apex (Figure 3). Three weeks after retreatment numbness was still present. After evaluation and discussion with the patient, in order to prevent permanent nerve damage, decision for extraction was made (Figure 4). After tooth extraction, a progressive healing appeared up to the complete resolution of neurological disturbance. Six months after tooth extraction, at the radiological examination, complete disappearance of the lesions was observed and implant rehabilitation was performed (Figure 5).

**Figure 6:**
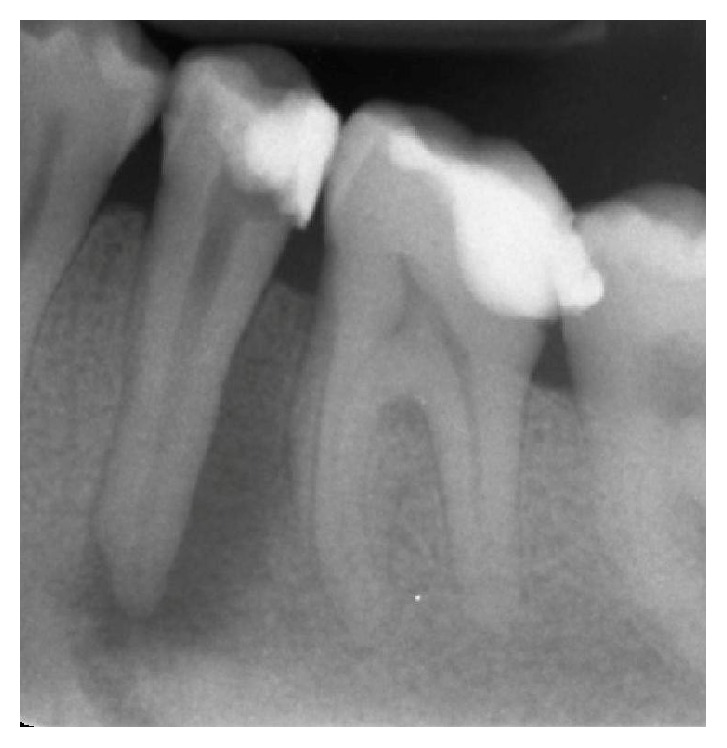
Mandibular left first premolar with a wide lesion in proximity to the mental foramen.

**Figure 7:**
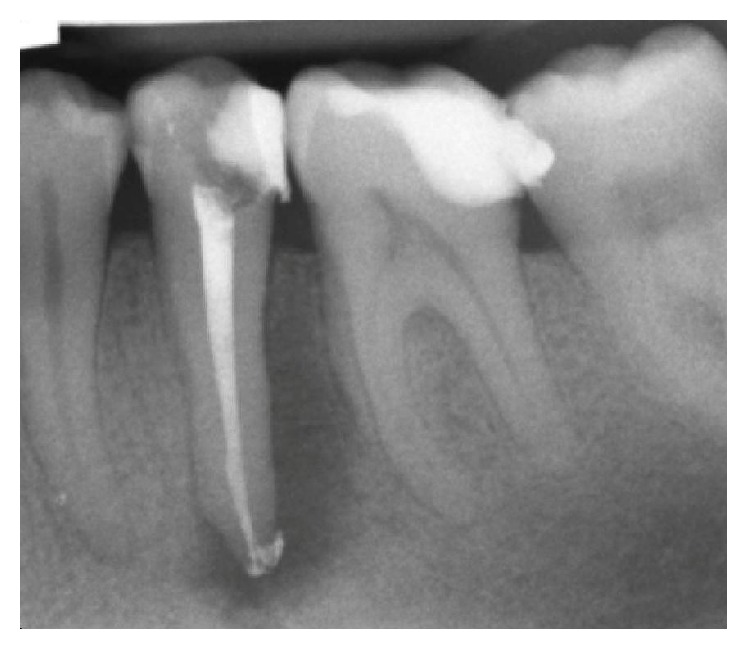
Radiographic control after endodontic treatment.

**Figure 8:**
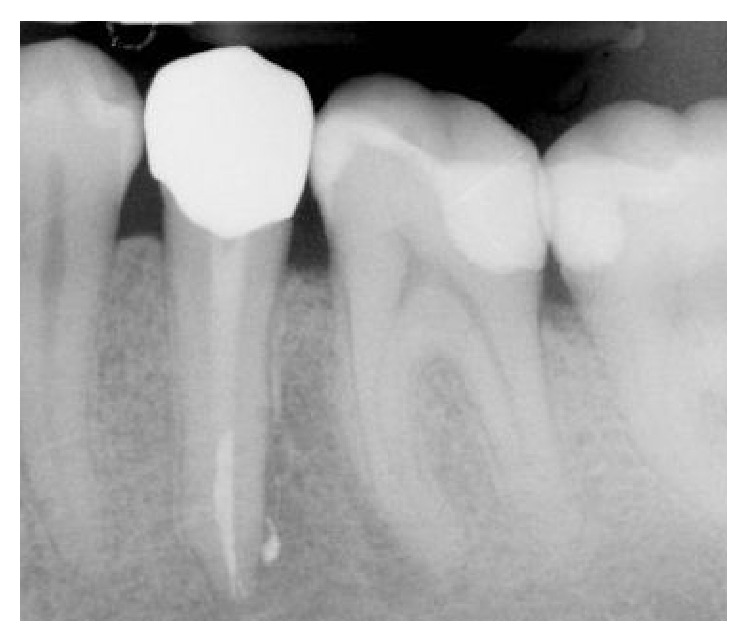
Radiographic control at one-year follow-up.

*Case  2*. At the initial endodontic appointment, a 61-year-old male patient arrived presenting hemimandibular pain and numbness on the left half of the lip of a 3-day evolution. The patient said that he was previously visited by his general doctor who prescribed him antibiotic therapy (amoxicillin and clavulanic acid 1 gr, 1 tablet every 8 hours for 7 days). The medical history was uneventful. Intraoral examination showed the mandibular left first premolar presented a wide composite filling. The premolar was not sensitive to percussion or palpation and gave no response to cold test. A periapical radiograph was taken and showed a wide lesion in proximity to the mental foramen (Figure 6). It was decided to immediately start the endodontic treatment. Upon opening the tooth, it was found that the pulp was necrotic. There was one canal, and it was completely instrumented and irrigated with sodium hypochlorite. A dry cotton pellet was inserted in the canal and Telio (Ivoclar Vivadent AG, Liechtenstein) was positioned as the provisional restoration. It was suggested to the patient that he integrate his antibiotic therapy with Metronidazole (Flagyl, Pfizer, 500 mg every 8 hours for 7 days). One week later, the patient returned for his appointment. During the week, he had no pain or swelling and a decrease in the paresthesia (a tingling of the lip) was observed. Considering the presence of exudate in the canal, it was not possible to complete the endodontic treatment. At the third appointment, the root canal was finally filled with gutta-percha and sealer (EndoRez; Ultradent Products Inc., Salt Lake City, UT) (Figure 7). One month later, the paresthesia had completely resolved and the patient was asymptomatic; therefore prosthetic finalization was performed. The 1-year follow-up radiograph showed clear sign of healing (Figure 8).

## 3. Discussion

The endodontic-related paresthesia is a rare complication that can be related to iatrogenic or pathologic causes. Considering iatrogenic factors, paresthesia might be caused by overinstrumentation and/or overfill or the passage of endodontic material into the vicinity of the inferior alveolar nerve and it is most frequently associated with a clinical error. In the cases described, the paresthesia is not directly related to an incorrect endodontic therapy but it is a consequence of the presence of a periapical lesion. In the cases reported, the paresthesia can be the result of a combination of the following etiopathogenic mechanisms.

*(1) Mechanical Pressure*. In particular, the expanding infectious process can cause pressure on the nerve fibers. The pressure induces the paresthesia.

*(2) Microbial Products*. It was demonstrated that the microbial products of certain microbes (gram negative bacteria) can breach the perineurium with resultant nerve bundle deterioration and impaired conduction [8].

 These observations were also described by Morse [9] who reviewed a series of cases in which the paresthesia occurred before the endodontic treatment. In particular, he reported three articles in which six teeth with necrotic pulps and periapical lesions determined a paresthesia. In all the cases periapical lesions were associated with mandibular teeth and, in detail, 2 of the six teeth were premolar whereas the others were molars. A case of paresthesia associated with an inflammatory cyst in correspondence with teeth 4.4 and 4.5 was also described by Jerjes et al. [10]. As reported in literature, because of the proximity of the mental foramen, the mental nerve is usually affected by endodontic-related complications in mandibular premolars [11]. Considering the mandibular molars, high attention should also be paid to the distance between the apices and the mandibular canal. In the study by Tilotta-Yasukawa et al. [12], it is reported that the distance varies between 1 and 4 mm in case of the first mandibular molar and it is less than 1 mm with the second and third mandibular molars. For this reason, an infectious process that originates from the apices of the first or second mandibular molar may very quickly involve the inferior alveolar nerve even if the periapical lesion size is small.

The recovery potential of the nerve depends on the extent of the damage and rapidity of cause removal [13]. The majority of iatrogenic damage cases are treated by pharmacologic therapy. In some cases, surgical exploration is required to remove the foreign material from the periapical area as soon as possible (within 48 hours) or when necessary, extraction of the tooth is required. However, in these cases of iatrogenic injuries, the best treatment is prevention. In fact, complications might be prevented by careful preoperative examination, good quality radiographs and good instrumentation, and irrigation and obturation techniques.

In case of endodontic-related paresthesia associated with a local infection, the prevention has a marginal role, because generally paresthesia occurs before the endodontic treatment, whereas great importance is related to the treatment options. Paresthesia resulting from periapical lesions usually subsides through elimination of infection by root canal treatment. In general, the disinfection of the root canal system and the chemomechanical instrumentation are the major factors contributing to the healing of the endodontic lesion and to the favorable outcome. During the appropriate nonsurgical endodontic treatment, pharmacologic therapy can be helpful. In particular, drugs such as antibiotics, nonsteroidal anti-inflammatory drugs and corticosteroids, proteolytic enzymes, and vitamin C are used in order to reduce the effects of ischemia and to control inflammation, edema, hematoma, or infection. Moreover, some authors suggest prescribing drugs that stimulate the reparative phase. These drugs include topical steroids, cocarnitine, somatotropic hormone, nerve growth factor, vitamin E, vasodilators, and ozone which improves the activity of red corpuscles and increases tissue oxygenation, as described in literature [2, 14]. Another treatment solution is represented by surgical intervention. In particular, according to Zuniga [15], it is reported that if there are no signs of enhancement in an early phase (within 3 months after injury), the immediate extraction of the tooth is the treatment of choice in order to prevent irreversible paresthesia [16] because better treatment outcomes are achieved if nerve paresthesia is treated as early as possible. On the contrary, Gregg [17] stated that because most IAN injuries are known to resolve spontaneously, there is no conclusive evidence that early intervention is better than delayed nonsurgical management (more than 3 months after injury). In the first case presented, after careful consideration and discussion with the patient, the final treatment planning was decided for the tooth extraction because no clinical sign of enhancement was observed. In fact, there seems to be a correlation between the duration of mechanical or chemical irritation and the risk that the paresthesia will become permanent [18].

## 4. Conclusion

Paresthesia resulting from local infection usually subsides through elimination of the infectious process by root canal treatment, pharmacologic therapy, or extraction. The patient must be informed of the nature and possible duration of paresthesia.
